# Plant-Based Dietary Indices in Relation to Nutrient and Food Group Intakes in Preschool-Aged Children

**DOI:** 10.3390/nu15214617

**Published:** 2023-10-31

**Authors:** Patricia F. C. Acosta, Olivia A. Landon, Zachary J. Ribau, Jess Haines, David W. L. Ma, Alison M. Duncan, on behalf of the Guelph Family Health Study

**Affiliations:** 1 Department of Human Health and Nutritional Sciences, University of Guelph, Guelph, ON N1G 2W1, Canada; pacosta@uoguelph.ca (P.F.C.A.); olandon@uoguelph.ca (O.A.L.); zribau@uoguelph.ca (Z.J.R.); davidma@uoguelph.ca (D.W.L.M.); 2 Department of Family Relations and Applied Nutrition, University of Guelph, Guelph, ON N1G 2W1, Canada; jhaines@uoguelph.ca

**Keywords:** dietary guidance, dietary assessment, plant-based dietary index, nutrient intakes, food group intakes, preschool-aged children

## Abstract

Dietary guidance promotes plant-based foods, yet minimal research has examined intake in children. This study examined plant-based food intake in preschool-aged children using plant-based dietary index (PDI) metrics and related these metrics to nutrient and food group intakes. Dietary data were collected from preschool-aged children (*n* = 283, 3.45 ± 1.22 years) from the Guelph Family Health Study at baseline using the Automated Self-Administered 24-Hour Dietary Assessment Tool. Food intake servings were assigned to 16 food groups for calculation of overall PDI (oPDI), healthful PDI (hPDI), and less healthful (lhPDI) scores and summarized into tertiles for energy-adjusted comparisons. For oPDI, participants in the highest vs. lowest tertile had higher intakes of nutrients and food groups to encourage (e.g., dietary fiber, fruits) as well as lower intakes of nutrients to encourage (e.g., calcium, vitamin D). For hPDI, participants in the highest vs. lowest tertile had higher intakes of nutrients and food groups to encourage and lower intakes of those to limit (e.g., saturated fat, sweets and desserts). For lhPDI, participants in the highest vs. lowest tertile had higher intakes of nutrients and food groups to limit and lower intakes of those to encourage. These results can inform dietetic practice for dietary guidance that promotes plant-based foods in children.

## 1. Introduction

Dietary guidelines from numerous countries have shifted towards the promotion of sustainable dietary patterns that include higher intakes of plant-based foods [1,2,3,4,5]. Higher intakes of plant-based foods, including nuts, seeds, and legumes, fruits, vegetables, and whole grains, have been related to improved nutrient intake, including higher dietary fiber, micronutrients, and unsaturated fat [6], as well as improved diet quality [7]. Higher intakes of plant-based foods have also been related to improved health outcomes, including lower risk of cardiovascular disease [8,9,10,11], type 2 diabetes [12,13], breast cancer [14,15,16], and all-cause mortality [17,18,19,20]. These improvements support the dietary guidance and rationalize the measurement of plant-based food intake in various population segments.

Plant-based food intake can be measured using the plant-based dietary index (PDI), as first described by Satija et al. [12]. The PDI is designed to examine the dietary intake distribution of plant- and animal-based foods using a system that assigns positive or reverse scores to plant foods and reverse scores to animal foods [12]. The metrics include an overall PDI as well as a healthful PDI and an unhealthful PDI that reflect intake of plant-based foods that are healthy (e.g., fruits, whole grains) or less healthy (e.g., sweets and desserts, sugar sweetened beverages (SSBs)) [12]. The PDI metrics have been examined in relation to chronic disease risk in adults in various locations including North America [8,12,21,22], Europe [23,24], and Korea [10,18,25]. There is rationale to also examine PDI metrics in children, particularly since dietary habits established in young childhood can be associated with health outcomes [26] and persist into adulthood [27]. Therefore, the purpose of the current study was to examine intake of plant-based foods using the PDI metrics and relate them to nutrient and food group intakes in preschool-aged children participating in the Guelph Family Health Study (GFHS).

## 2. Materials and Methods

### 2.1. Study Design and Participant Screening

The current study used baseline dietary assessment data from the GFHS, an ongoing cohort study examining the effects of home-based lifestyle interventions on obesity prevention in families with young children. The study was approved by the University of Guelph Research Ethics Board (REB#17-07-003) and registered on ClinicalTrials.gov (NCT02939261). All parents provided written consent and, when possible, children provided verbal assent.

Participants included children between 1.5 and 5 years who were in GFHS families. Families were eligible if they resided in Guelph, Ontario, or surrounding areas, had a parent who could respond to questionnaires in English, and did not have a participating child(ren) with a severe health condition.

Of the 293 children who met the inclusionary criteria, 10 were excluded due to a missing dietary assessment (*n* = 1) or errors in their dietary assessment entries (*n* = 6), including an implausible energy intake (<500 kcal/day) (*n* = 3).

### 2.2. Anthropometric Measurements

Height was measured using a stadiometer (ShorrBoard, Weight and Measure, LLC., Olney, MD, USA). Body weight was measured using an electronic scale (BOD POD™, COSMED, Concord, CA, USA). Body mass index (BMI) z-scores were calculated using World Health Organization Anthro software (version 3.2.2, Geneva, Switzerland, 2011).

### 2.3. Dietary Assessment

Dietary assessment was completed by the participant’s parent for a 24 h period using the National Cancer Institute’s web-based Automated Self-Administered 24 h (ASA24) Dietary Assessment Tool, version ASA24-Canada-2016, adapted to reflect the Canadian food supply, portion sizes, and nutrient composition. ASA24 includes multiple prompts for participants to facilitate accurate data entry and has been validated for use in children [28]. ASA24-Canada analyzes the dietary data using the Canadian Nutrient File and a Health Canada recipe database along with the United States Food and Nutrient Database for Dietary Studies (FNDDS) and the Food Patterns Equivalents Database (FPED). These databases enable ASA24-Canada to output a summary of the food descriptions, energy and nutrient intakes, and United States Department of Agriculture (USDA) Food Pattern components.

### 2.4. Plant-Based Dietary Index (PDI) Scoring

**Figure 1:**
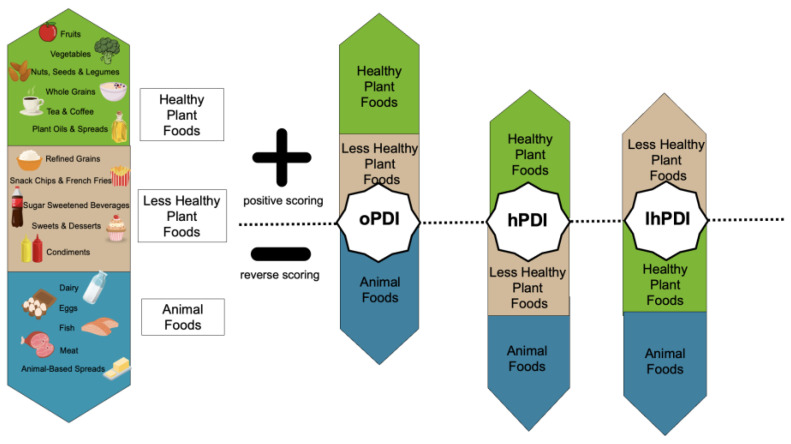
Summary of the PDI metrics scoring process. Abbreviations used: oPDI, overall plant-based dietary index; hPDI, healthful plant-based dietary index; lhPDI, less healthful plant-based dietary index. Created with Canva, adapted with permission from Sarah E. Jarvis.

PDI scores were computed for overall PDI (oPDI), healthful PDI (hPDI), and less healthful PDI (lhPDI), adapted from Satija et al., 2016 [12]. Food intakes from the ASA24 results output were converted from grams to servings using Health Canada’s Table of Reference Amounts for Food [29]. Food descriptions reported as mixed dishes were disaggregated and quantified using information from the detailed ASA24 responses and the ASA24 food group variables, followed by conversion to grams using data from the FPED. Food servings were then assigned to 1 of 16 food groups categorized as healthy plant foods (whole grains; fruits; vegetables; nuts, seeds, and legumes; tea and coffee; plant oils and spreads), less healthy plant foods (refined grains; snack chips and French fries; SSB; sweets and desserts; condiments), or animal foods (dairy; eggs; fish; meat; animal-based spreads) (Figure 1). Servings in each food group were totalled for each participant and summarized into food group serving intake quintiles (Q) across all participants. PDI scores were then computed for each participant by relating their total intake of food group servings to the food group serving intake Qs. For oPDI, scores of 1 were assigned to all plant food group serving intakes that were in Q1 and scores of 5 were assigned to all plant food group serving intakes that were in Q5 (positive scoring such that Q1 = 1, Q2 = 2, Q3 = 3, Q4 = 4, Q5 = 5), and the opposite approach was completed for animal food group serving intakes (reverse scoring such that Q1 = 5, Q2 = 4, Q3 = 3, Q4 = 2, Q5 = 1) (Figure 1). For hPDI, positive scoring was completed for healthy plant food group serving intakes and reverse scoring was completed for less healthy plant food and animal food group serving intakes (Figure 1). For lhPDI, positive scoring was completed for less healthy plant food group serving intakes and reverse scoring was completed for healthy plant food and animal food group serving intakes (Figure 1). Scores were summed within a participant for oPDI, hPDI, and lhPDI with theoretical ranges of 16 to 80.

### 2.5. Data and Statistical Analysis

All data were analyzed using the Statistical Analysis System (SAS Institute Inc., Version 9.4, Cary, NC, USA) with *p* < 0.05 considered significant. All dietary data were examined for normality using box plots and stem-leaf diagrams and log-transformed where appropriate. Summary statistics were generated for sex, BMI z-score, and PDI scores, and tertiles were computed for PDI scores. Nutrient and food group intakes were compared among oPDI, hPDI, and lhPDI tertiles using the GENMOD procedure (to implement the generalized estimating equation approach to control for correlated outcomes among siblings), adjusted for energy intake, and followed by a Tukey’s test for multiple comparisons.

## 3. Results

### 3.1. Participant Characteristics

Participants included 148 girls and 135 boys who had a mean ± SD age of 3.45 ± 1.22 years and BMI z-score of 0.58 ± 0.98.

### 3.2. oPDI Scores in Relation to Nutrient and Food Group Intakes

**Figure 2:**
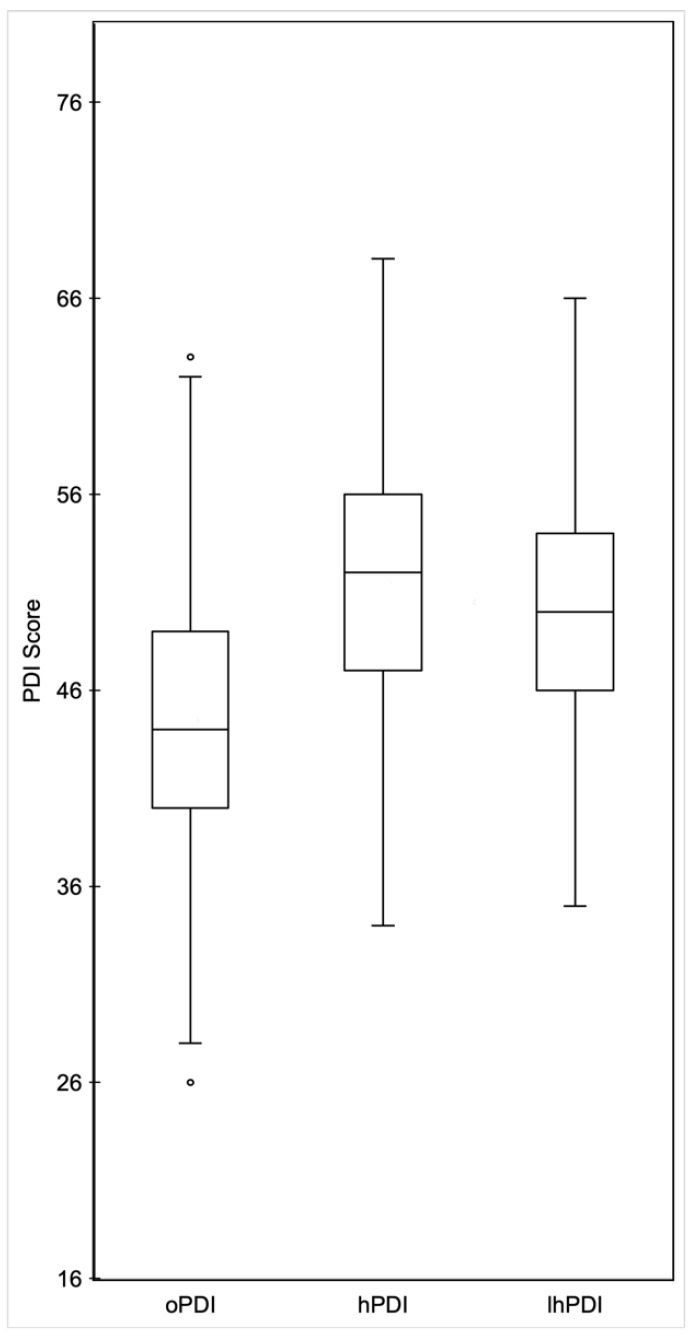
PDI metric box plots. Distribution of PDI scores of children for oPDI, hPDI, and lhPDI. Abbreviations: PDI, plant-based dietary index; oPDI, overall plant-based dietary index; hPDI, healthful plant-based dietary index; lhPDI, less healthful plant-based dietary index.

The median oPDI score was 42 with a range of 26–63 (Figure 2). Tertiles for oPDI were 26–41 (*n* = 105) for tertile 1, 42–47 (*n* = 87) for tertile 2, and 48–63 (*n* = 91) for tertile 3.

**Figure 3:**
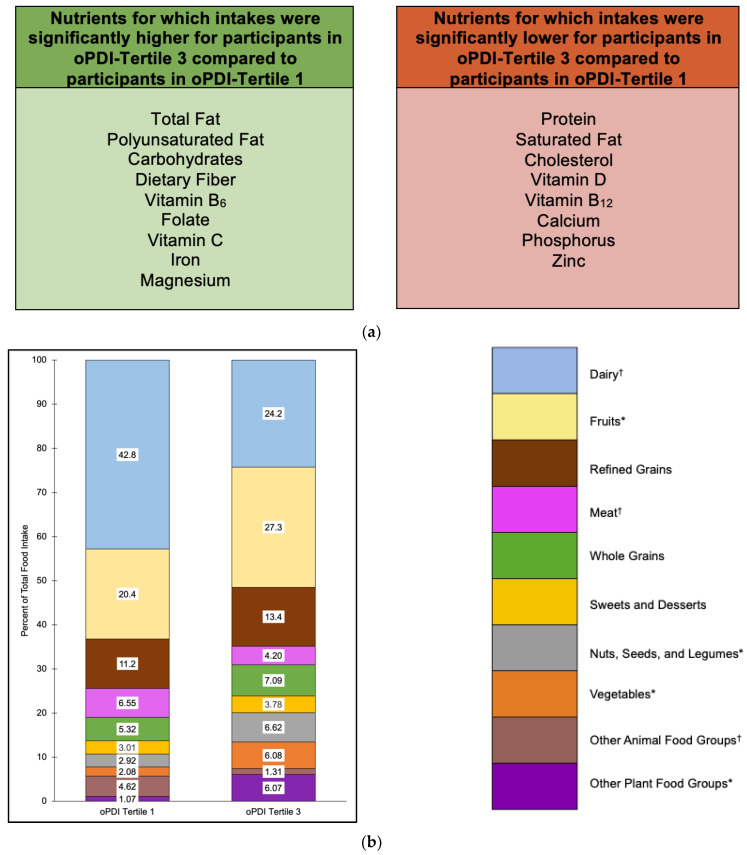
Nutrient intakes (**a**) and food group proportional intakes (**b**) of participants in oPDI tertile 3 compared to oPDI tertile 1. Values within each food group bar segment are percent of total food intake. Other animal food groups and other plant food groups are combinations of food groups with intakes ≤3% of total food intake for both tertiles 1 and 3. Intakes for food groups in the legend with * were significantly higher for oPDI tertile 3 compared to oPDI tertile 1 (within the other plant food groups, this refers to snack chips and French fries, sugar-sweetened beverages, and condiments). Intakes for food groups in the legend with ^†^ were significantly lower for oPDI tertile 3 compared to oPDI tertile 1 (within the other animal food groups, this refers to eggs and animal-based spreads). Abbreviations: oPDI, overall plant-based dietary index.

Nutrient intakes that were significantly higher for participants in oPDI tertile 3 compared to tertile 1 included total fat (*p* = 0.0004), polyunsaturated fat (*p* = 0.03), carbohydrates (*p* < 0.0001), dietary fiber (*p* < 0.0001), vitamin B_6_ (*p* = 0.03), folate (*p* = 0.005), vitamin C (*p* = 0.0002), iron (*p* < 0.0001), and magnesium (*p* < 0.0001) (Figure 3a and Table S1, Supplementary Data). Nutrient intakes that were significantly lower for participants in oPDI tertile 3 compared to tertile 1 included protein (*p* < 0.0001), saturated fat (*p* < 0.0001), cholesterol (*p* < 0.0001), vitamin D (*p* < 0.0001), vitamin B_12_ (*p* < 0.0001), calcium (*p* < 0.0001), phosphorus (*p* < 0.0001), and zinc (*p* = 0.005) (Figure 3a and Table S1, Supplementary Data).

Food groups that contributed the highest proportions of food intake for oPDI tertiles 1 and 3 included dairy (42.8% and 24.2%, respectively), fruits (20.4% and 27.3%, respectively), and refined grains (11.2% and 13.4%, respectively) (Figure 3b). Food groups that contributed the lowest proportions of food intake accounted for ≤3% of total food intake for oPDI tertiles 1 and 3 and included SSB (0% and 2.42%, respectively), snack chips and French fries (0.34% and 1.30%, respectively), condiments (0.30% and 1.49%, respectively), plant oils and spreads (0.22% and 0.43%, respectively), tea and coffee (0.21% and 0.43%, respectively), eggs (2.97% and 0.92%, respectively), animal-based spreads (0.91% and 0.28%, respectively), and fish (0.74% and 0.11%, respectively) (Figure 3b).

Food group proportional intakes that were significantly higher for participants in oPDI tertile 3 compared to tertile 1 included fruits (*p* = 0.003); nuts, seeds, and legumes (*p* = 0.01); vegetables (*p* = 0.002); snack chips and French fries (*p* = 0.02), SSB (*p* = 0.03); and condiments (*p* < 0.0001) (Figure 3b). Food group proportional intakes that were significantly lower for participants in oPDI tertile 3 compared to tertile 1 included dairy (*p* < 0.0001), meat (*p* = 0.02), eggs (*p* = 0.0002), and animal-based spreads (*p* < 0.0001) (Figure 3b).

### 3.3. hPDI Scores in Relation to Nutrient and Food Group Intakes

The median hPDI score was 52 with a range of 34–68 (Figure 2). Tertiles for hPDI were 34–49 (*n* = 98) for tertile 1, 50–55 (*n* = 97) for tertile 2 and 56–68 (*n* = 88) for tertile 3.

**Figure 4:**
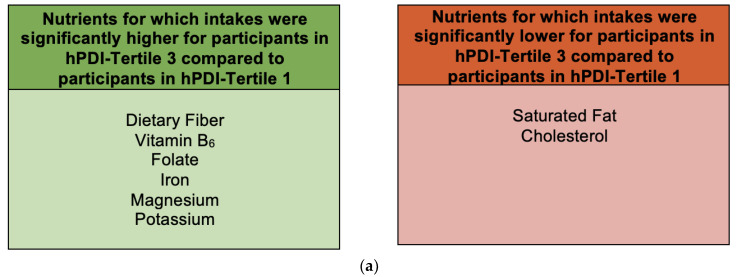

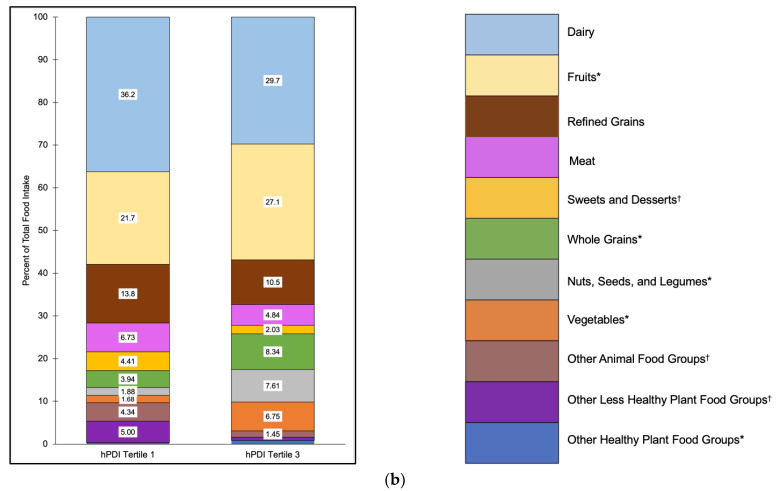
Nutrient intakes (**a**) and food group proportional intakes (**b**) of participants in hPDI tertile 3 compared to hPDI tertile 1. Values within each food group bar segment are percent of total food intake. Other animal food groups, other less healthy plant food groups, and other healthy plant food groups are combinations of food groups with intakes ≤3% of total food intake for both tertiles 1 and 3. Intakes for food groups in the legend with * were significantly higher for hPDI tertile 3 compared to tertile 1 (within the other healthy plant food groups, this refers to plant oils and spreads). Intakes for food groups in the legend with ^†^ were significantly lower for hPDI tertile 3 compared to hPDI tertile 1 (within the other animal food groups, this refers to eggs and animal-based spreads, and within the other less healthy plant food groups, this refers to snack chips and French fries, sugar-sweetened beverages, and condiments). Abbreviations: hPDI, healthful plant-based dietary index.

Nutrient intakes that were significantly higher for participants in hPDI tertile 3 compared to tertile 1 included dietary fiber (*p* < 0.0001), vitamin B_6_ (*p* = 0.009), folate (*p* = 0.004), iron (*p* = 0.004), magnesium (*p* < 0.0001), and potassium (*p* < 0.0001) (Figure 4a and Table S2, Supplementary Data). Nutrient intakes that were significantly lower for participants in hPDI tertile 3 compared to tertile 1 included saturated fat (*p* = 0.003) and cholesterol (*p* = 0.0002) (Figure 4a and Table S2, Supplementary Data).

Food groups that contributed the highest proportions of food intake for hPDI tertiles 1 and 3 included dairy (36.2% and 29.7%, respectively), fruits (21.7% and 27.1%, respectively), and refined grains (13.8% and 10.5%, respectively) (Figure 4b). Food groups that contributed the lowest proportions of food intake accounted for ≤3% of total food intake for hPDI tertiles 1 and 3 and included SSB (2.05% and 0.16%, respectively), snack chips and French fries (1.70% and 0.12%, respectively), condiments (1.25% and 0.49%, respectively), tea and coffee (0.26% and 0.48%, respectively), plant oils and spreads (0.08% and 0.39%, respectively), eggs (2.47% and 0.94%, respectively), animal-based spreads (0.96% and 0.29%, respectively), and fish (0.91% and 0.22%, respectively) (Figure 4b).

Food group proportional intakes that were significantly higher for participants in hPDI tertile 3 compared to tertile 1 included fruits (*p* = 0.03), whole grains (*p* = 0.0004), nuts, seeds, and legumes (*p* < 0.0001), vegetables (*p* = 0.0005), and plant oils and spreads (*p* < 0.0001) (Figure 4b). Food group proportional intakes that were significantly lower for participants in hPDI tertile 3 compared to tertile 1 included sweets and desserts (*p* = 0.002), eggs (*p* = 0.008), animal-based spreads (*p* < 0.0001), snack chips and French fries (*p* < 0.0001), SSB (*p* = 0.03), and condiments (*p* = 0.02) (Figure 4b).

### 3.4. lhPDI Scores in Relation to Nutrient and Food Group Intakes

The median lhPDI score was 50 with a range of 35–66 (Figure 2). Tertiles for oPDI were 35–47 (*n* = 101) for tertile 1, 48–52 (*n* = 86) for tertile 2, and 53–66 (*n* = 96) for tertile 3.

**Figure 5:**
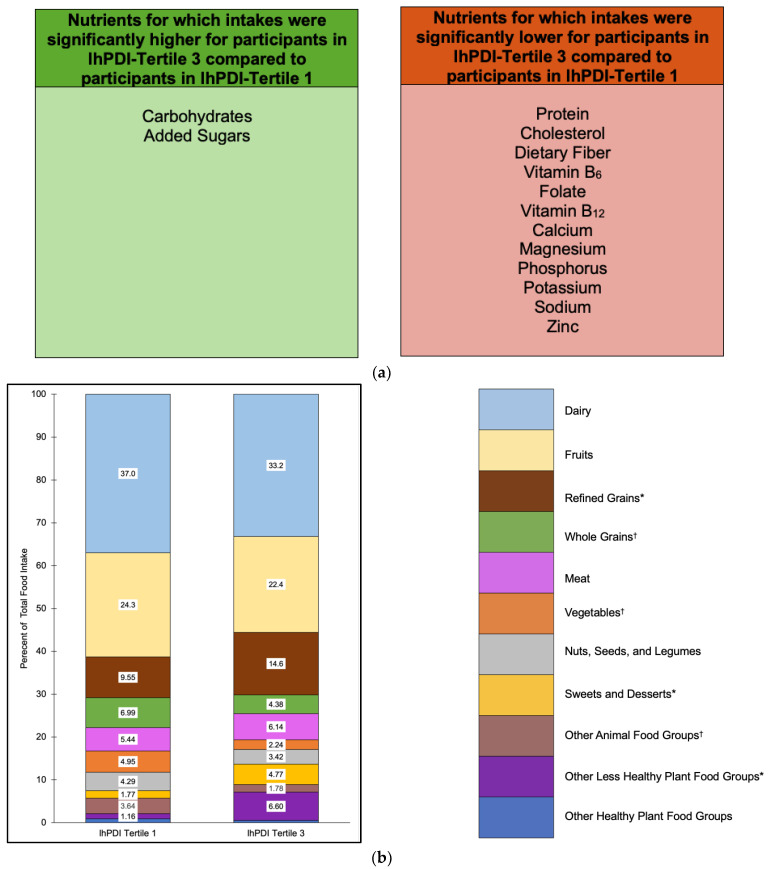
Nutrient intakes (**a**) and food group proportional intakes (**b**) of participants in lhPDI tertile 3 compared to lhPDI tertile 1. Values within each food group bar segment are percents of total food intake. Other animal food groups, other less healthy plant food groups, and other healthy plant food groups are combinations of food groups with intakes ≤3% for both tertiles 1 and 3. Intakes for food groups in the legend with * were significantly higher for lhPDI tertile 3 compared to lhPDI tertile 1 (within the other less healthy plant food groups, this refers to snack chips and French fries, and condiments). Intakes for food groups in the legend with ^†^ were significantly lower for lhPDI tertile 3 compared to lhPDI tertile 1 (within the other animal food groups, this refers to fish). Abbreviations: lhPDI, less healthful plant-based dietary index.

Nutrient intakes that were significantly higher for participants in lhPDI tertile 3 compared to tertile 1 included carbohydrates (*p* = 0.001) and added sugars (*p* < 0.0001) (Figure 5a and Table S2, Supplementary Data). Nutrient intakes that were significantly lower for participants in lhPDI tertile 3 compared to tertile 1 included protein (*p* < 0.0001), cholesterol (*p* < 0.0001), dietary fiber (*p* < 0.0001), vitamin B_6_ (*p* = 0.002), folate (*p* = 0.01), vitamin B_12_ (*p* = 0.003), calcium (*p* = 0.0005), magnesium (*p* < 0.0001), phosphorus (*p* < 0.0001), potassium (*p* < 0.0001), sodium (*p* = 0.04), and zinc (*p* < 0.0001) (Figure 5a and Table S2, Supplementary Data).

Food groups that contributed the highest proportions of food intake for lhPDI tertiles 1 and 3 included dairy (37.0% and 33.2%, respectively), fruits (24.3% and 22.4%, respectively), and refined grains (9.55% and 14.6%, respectively) (Figure 5b). Food groups that contributed the lowest proportions of food intake accounted for ≤3% of total food intake for lhPDI tertiles 1 and 3 and included SSB (0.69% and 3.02%, respectively), snack chips and French fries (0.13% and 2.17%, respectively), condiments (0.34% and 1.41%, respectively), tea and coffee (0.59% and 0.25%, respectively), plant oils and spreads (0.34% and 0.27%, respectively), eggs (2.32% and 1.23%, respectively), animal-based spreads (0.63% and 0.49%, respectively), and fish (0.69% and 0.06%, respectively) (Figure 5b).

Food group proportional intakes that were significantly higher for participants in lhPDI tertile 3 compared to tertile 1 included refined grains (*p* = 0.0009), sweets and desserts (*p* < 0.0001), snack chips and French fries (*p* < 0.0001), and condiments (*p* = 0.0003) (Figure 5b). Food group proportional intakes that were significantly lower for participants in lhPDI tertile 3 compared to tertile 1 included whole grains (*p* = 0.02), vegetables (*p* = 0.03), and fish (*p* = 0.008) (Figure 5b).

## 4. Discussion

The current study examined plant-based food intake in a sample of 283 preschool-aged children who were participating in the GFHS. Dietary assessment was completed for a 24 h period by each child’s parent using the online-based ASA24. The itemized food intakes were converted to servings and categorized into 11 plant food groups or 5 animal food groups for calculation of oPDI scores. Plant food groups were further categorized into healthy or less healthy plant food groups for calculation of hPDI and lhPDI scores. The focus on dietary distribution of plant-based food intakes in young children is relevant as it is a critical stage for growth and development, with unique nutritional requirements [30]. Gaining insights into the nutritional implications of plant-based foods in children’s diets is pertinent as evidence demonstrates that childhood dietary habits can persist into adulthood [27]. As such, the current study examined the PDI metric scores and related them to nutrient and food group intakes in a sample of preschool-aged children.

The current study’s focus on young children in its examination of plant-based food intake adds diversity to the participants that have been studied in this literature. Adults have been the focus of most of the previous studies of plant-based food intake, which have related PDI metrics to various health conditions [12,14,19,25]. The need for research conducting thorough examinations of plant-based food intake in children is important since childhood dietary habits can continue through into adulthood [27] and relate to health outcomes [26]. Overall, since dietary guidance includes children, the current study’s participant sample adds necessary diversity to the plant-based food intake literature.

The range of oPDI scores in the current study is 26–63 out of a theoretical range of 16–80. When oPDI scores are summarized into tertiles, the results show that participants who have higher overall plant intake (oPDI-tertile 3) have higher intakes of nutrients to encourage (polyunsaturated fat, dietary fiber, vitamin B_6_, folate, vitamin C, iron, magnesium), and, except for saturated fat and cholesterol, also have lower intakes of nutrients to encourage (protein, vitamin D, vitamin B_12_, calcium, phosphorus, zinc). The food group intakes accounted for in these nutrient intakes show that participants who have higher oPDI have higher intakes of fruits, vegetables, nuts, seeds, and legumes, and lower intakes of dairy and meat. The majority of PDI studies have been conducted in adults and focus on health outcomes; however, Chen et al. studied children aged 6–9 years living in China [31]. Their food group results show that participants with higher overall plant intake have higher intakes of healthy plant foods and also some less healthy plant foods, although statistical comparisons are not completed and nutrient intakes are not reported [31]. Other PDI studies that have examined nutrient intakes report higher PDI scores in relation to higher intakes of carbohydrates [32,33], polyunsaturated fat [33], dietary fiber and vitamin B_6_ [32,33], folate [32], vitamin C [33,34], and magnesium [32,33], and lower intakes of protein [15,21,33,34], total fat [15,33,34], saturated fat [15,33,34], cholesterol, vitamin B_6_, vitamin B_12_, calcium, and magnesium [15,33], although all of these studies were conducted in Iranian adults, except for one that was in South Korean adults [21]. Collectively, these studies support a role for plant foods in promoting the intake of nutrients beneficial for health, but also demonstrate that other nutrients beneficial for health can be lower with varying intakes of certain plant foods. These results argue for a comprehensive dietary approach that includes a diversity of plant foods to support optimal nutrient intake.

The hPDI metric further examines plant food intake by considering the intake of plant foods that are considered healthy. The range of hPDI scores in the current study is 34–68 out of a theoretical range of 16–80. Participants who have higher intakes of healthy plant foods (hPDI-tertile 3) have higher intakes of nutrients to encourage (dietary fiber, vitamin B_6_, folate, iron, magnesium, potassium) and lower intakes of a nutrient to limit (saturated fat). These results correspond with higher food group intakes, including higher intakes of healthy plant foods (fruits, whole grains, nuts, seeds, and legumes, vegetables, and plant oils and spreads). Previous PDI studies also report higher hPDI scores in relation to higher intakes of dietary fiber [9,15,33,35], vitamin B_6_ [15,33], folate [9,15,21], iron [21], and magnesium [9,15,21], and lower intakes of saturated fat [9,15,21,33,35], although all of these studies were conducted in adults. These results demonstrate that higher hPDI scores reflect a diet high in food groups to encourage and contribute to optimal nutrient intakes by promoting increased intakes of nutrients to encourage and lower intakes of nutrients to limit.

The lhPDI metric also further examines plant food intake by considering the intake of plant foods that are considered less healthy. The range of lhPDI scores is 35–66 out of a theoretical range of 16–80. Participants who have higher intakes of less healthy plant foods (lhPDI-tertile 3) have higher intakes of nutrients and food groups to limit (added sugars, sweets and desserts, and snack chips and French Fries) and lower intakes of nutrients and food groups to encourage (protein, dietary fiber, several micronutrients, whole grains, vegetables, and fish). These results are consistent with previous studies, all conducted in adults, that also report higher lhPDI scores in relation to higher intakes of added sugars [35], and lower intakes of protein [21,33], dietary fiber [15,21,33,35], and micronutrients, including vitamin B_6_ [15,33], folate [15,21], calcium, magnesium, and potassium [21,33]. These findings highlight the consideration of nutritional quality when relating plant food intake to nutrient intakes. A higher intake of plant foods may not always be consistent with higher intakes of nutrients and food groups to encourage, which rationalizes the inclusion of the hPDI and lhPDI metrics in the dietary assessment of plant food intake.

In this study sample of preschool-aged children, dairy, fruits, and refined grains were the most frequently consumed food groups, accounting for >60% of total food intake, regardless of PDI metric or tertile. These food group intake results are consistent with previous studies that report children aged 2–6 years in China most frequently consume cereals, dairy, and fruits [36], and children aged 2–3 years in the United States consume milk and fruit at least once daily [37]. Dairy foods are nutrient-dense, providing high-quality protein and many micronutrients, including calcium and vitamin D to support growth and development in children [38]. Fruits also provide multiple micronutrients and can be high in dietary fiber, which can all support health [39]. Refined grains can be a source of multiple shortfall micronutrients, including folic acid and iron, to contribute toward nutrient adequacy [40]. Nonetheless, dietary intake can always be improved with more variety and increased intakes of certain foods such as vegetables, and nuts, seeds, and legumes, which can contribute several nutrients to encourage, including dietary fiber, polyunsaturated fat, monounsaturated fat, and multiple micronutrients [41,42]. These findings demonstrate a need for greater diversity in children’s diets to promote nutrient intake adequacy and foster lifelong healthy dietary habits.

The current study is limited in its use of a single, self-reported 24 h dietary recall, which has inherent recall bias and/or measurement error. A strength of this study is its focus on children, who have been less studied, despite evidence that eating habits at a young age can persist into adulthood [27]. Another strength is the rigorous process employed to disaggregate mixed dishes into individual foods, which contributed to an increased accuracy of food group classification and subsequent PDI metric scoring.

## 5. Conclusions

In conclusion, the current study examined plant food intake in a sample of 283 preschool-aged children using PDI metrics. The results show that participants who have higher intakes of plant foods (oPDI) have higher intakes of nutrients and food groups to encourage (e.g., dietary fiber, fruits) but also lower intakes of nutrients to encourage (e.g., calcium, vitamin D). When plant food intake is further examined according to healthfulness, results predictably show that participants who have higher intakes of healthy plant foods (hPDI) have higher intakes of nutrients and food groups to encourage (e.g., dietary fiber, fruits), while participants who have higher intakes of less healthy plant foods (lhPDI) have higher intakes of nutrients and food groups to limit (e.g., added sugars and snack chips and French fries). These results provide evidence of the types of plant foods that preschool-aged children are consuming, and support directions for dietetic practice. Future research can include examinations of PDI scores in relation to health outcomes in children and effects of interventions on PDI scores in children. Overall, the results of this study can contribute toward the development of nutritional strategies that facilitate plant food intake in children.

## Data Availability

Interested researchers can contact GFHS investigators to explore data availability in alignment with the University of Guelph Research Ethics Board.

## Supplementary Materials

The following supporting information can be downloaded at https://www.mdpi.com/2072-6643/15/21/4617/s1: Table S1: Nutrient intakes by oPDI tertiles in preschool-aged children; Table S2: Nutrient intakes by hPDI and lhPDI tertiles in preschool-aged children.
