# Correction: Acosta et al. Plant-Based Dietary Indices in Relation to Nutrient and Food Group Intakes in Preschool-Aged Children. *Nutrients* 2023, *15*, 4617

**DOI:** 10.3390/nu16081186

**Published:** 2024-04-17

**Authors:** Patricia F. C. Acosta, Olivia A. Landon, Zachary J. Ribau, Jess Haines, David W. L. Ma, Alison M. Duncan, on behalf of the Guelph Family Health Study

**Affiliations:** 1 Department of Human Health and Nutritional Sciences, University of Guelph, Guelph, ON N1G 2W1, Canada; pacosta@uoguelph.ca (P.F.C.A.);; 2 Department of Family Relations and Applied Nutrition, University of Guelph, Guelph, ON N1G 2W1, Canada

## Figure Legend

In the original publication [1], there was a mistake in the caption for Figure 1, as published. The caption for Figure 1 is missing a permission statement. The corrected caption appears below.

**Figure 1.** Summary of the PDI metrics scoring process. Abbreviations used: oPDI, overall plant-based dietary index; hPDI, healthful plant-based dietary index; lhPDI, less healthful plant-based dietary index. Created with Canva, adapted with permission from Sarah E. Jarvis.

The authors state that the scientific conclusions are unaffected. This correction was approved by the academic editor. The original publication has also been updated.
