# Supplementary material for: Plant-Based Dietary Indices in Relation to Nutrient and Food Group Intakes in Preschool-Aged Children
Source: Nutrients. 2023 Oct 31;15(21):4617. doi: 10.3390/nu15214617 (PMC10647719; doi:10.3390/nu15214617)
Supplement: Supplementary file 1 [file nutrients-15-04617-s001.zip › nutrients-2669221-supplementary.pdf]

Table S1: Nutrient intakes by oPDI tertiles in preschool-aged children[1,2]

	**oPDI**		
	**Tertile 1** (26-41; *n* = 105)	**Tertile 2** (42-47; *n* = 87)	**Tertile 3** (48-63; *n* = 91)
Energy (kcal)	1301[a] ± 38.1	1459[b] ± 38.1	1502[b] ± 41.1
Protein[3] (g)	55.1[a] (51.6, 58.9)	54.6[b] (51.1, 58.4)	50.4[c] (47.2, 53.9)
Carbohydrates (g)	155.2[a] ± 5.50	187.1[b] ± 5.40	210.5[c] ± 5.79
Total Sugars (g)	74.0 ± 3.03	88.9 ± 3.50	91.6 ± 3.66
Added Sugars (g)	6.54 ± 0.53	8.40 ± 0.60	9.22 ± 0.66
Dietary Fiber (g)	12.0[a] ± 0.52	14.7[a] ± 0.55	19.1[b] ± 0.62
Total Fat (g)	51.5[a] ± 1.83	56.1[a] ± 2.20	53.4[b] ± 2.00
Saturated Fat (g)	21.0[a] ± 0.90	20.8[b] ± 0.92	17.1[c] ± 0.90
MUFA (g)	17.1 ± 0.63	19.4 ± 0.86	20.1 ± 0.78
PUFA (g)	8.35[a] ± 0.38	10.7[b] ± 0.51	11.3[b] ± 0.49

Cholesterol (mg)	234.7[a] ± 13.7	168.1[b] ± 9.70	138.4[c] ± 10.5
Vitamin B_6 (mg)	1.04 [a] ± 0.05	1.16[ab] ± 0.05	1.32[b] ± 0.05
Folate (μcg)	207.1[a] ± 8.33	236.3[ab] ± 10.1	269.9[b] ± 9.31
Vitamin B_{12} (μg)	3.64[a] ± 0.19	3.20[b] ± 0.18	2.53[c] ± 0.19
Vitamin C (mg)	57.7[a] ± 4.10	78.3[b] ± 5.74	90.9[b] ± 5.64
Vitamin D[3] (μg)	4.76[a] (4.24, 5.35)	3.67[b] (3.27, 4.12)	3.10[b] (2.71, 3.54)
Calcium (mg)	972.9[a] ± 43.5	951.5[a] ± 47.1	774.1[b] ± 39.5
Iron (mg)	7.51[a] ± 0.32	9.13[a] ± 0.32	10.8[b] ± 0.43
Magnesium[3] (mg)	188.7[a] (179.5, 198.3)	212.7[a] (199.0, 227.4)	242.3[b] (230.5, 254.6)
Phosphorus (mg)	1128[a] ± 42.5	1123[b] ± 42.5	1049[c] ± 36.2
Potassium (mg)	1866 ± 59.3	2062 ±80.4	2210 ± 65.8
Sodium (mg)	1898 ± 71.1	2136 ± 86.7	2117 ± 71.0
Zinc[3] (mg)	7.03[a] (6.58, 7.51)	7.54[ab] (7.05, 8.06)	6.82[b] (6.38, 7.29)

[1]Data are presented as means ± SE unless otherwise indicated.
[2]Values with different letter superscripts are significantly different from a comparison of tertiles using the GENMOD procedure (to implement the generalized estimating equation approach to control for correlated outcomes among siblings), adjusted for energy intake, and followed by a Tukey’s test for multiple comparisons.
[3]Values are geometric means (95% confidence intervals).
Abbreviations used: oPDI, overall plant-based dietary index; MUFA, monounsaturated fatty acids; PUFA, polyunsaturated fatty acids.

Table S2: Nutrient intakes by hPDI and lhPDI tertiles in preschool-aged children[1,2]

	hPDI			lhPDI		
	Tertile 1 (34-49; *n* = 98)	Tertile 2 (50-55; *n* = 97)	Tertile 3 (56-68; *n* = 88)	Tertile 1 (35-47; *n* = 101)	Tertile 2 (48-52; *n* = 86)	Tertile 3 (53-66; *n* = 96)
Energy (kcal)	1520[a] ± 40.9	1337[b] ± 36.4	1381[ab] ± 40.8	1474 ± 38.2	1360 ± 40.0	1400 ± 41.5
Protein[3] (g)	59.1 (56.3, 62.2)	50.4 (47.2, 53.9)	50.9 (47.6, 54.4)	62.2[a] (59.2, 65.4)	50.4[b] (47.2, 53.9)	47.9[b] (45.6, 50.4)
Carbohydrates (g)	191.2 ± 6.37	173.0 ± 5.61	184.1 ± 6.04	180.5[a] ± 5.62	177.9[b] ± 6.41	189.4[b] ± 6.19
Total Sugars (g)	88.1 ± 3.76	80.2 ± 3.16	84.3 ± 3.42	82.3 ± 3.20	83.5 ± 3.52	86.9 ± 3.70
Added Sugars (g)	9.00 ± 0.63	7.65 ± 0.57	7.18 ± 0.59	5.99[a] ± 0.48	8.0[b] ± 0.67	10.0[b] ± 0.60
Dietary Fiber (g)	13.3[a] ± 0.53	13.8[b] ± 0.60	18.6[c] ± 0.65	17.8[a] ± 0.63	14.6[b] ± 0.65	12.7[c] ± 0.52
Total Fat (g)	58.6 ± 1.98	50.4 ± 1.80	51.3 ± 2.14	57.8 ± 2.05	51.3 ± 1.80	51.1 ± 2.03

Saturated Fat (g)	22.6^{a} ± 1.03	19.2^{a} ± 0.79	17.1^{b} ± 0.83	21.4 ± 0.82	19.1 ± 0.97	18.5 ± 0.96
MUFA (g)	19.8 ± 0.66	17.4 ± 0.69	19.2 ± 0.92	20.4 ± 0.86	17.9 ± 0.65	17.8 ± 0.69
PUFA (g)	10.7 ± 0.46	9.12 ± 0.45	10.2 ± 0.50	10.4 ± 0.48	9.48 ± 0.47	10.0 ± 0.46
Cholesterol (mg)	230.0^{a} ± 12.5	173.1^{b} ± 10.1	142.4^{b} ± 13.3	226.7^{a} ± 13.7	166.3^{b} ± 11.9	152.7^{b} ± 10.1
Vitamin B_6 (mg)	1.16^{a} ± 0.05	1.08^{a} ± 0.05	1.27^{b} ± 0.05	1.30^{a} ± 0.05	1.11^{ab} ± 0.05	1.07^{b} ± 0.04
Folate (µcg)	233.9^{a} ± 8.85	229.5^{b} ± 10.2	246.5^{b} ± 9.53	263.0^{a} ± 10.5	220.3^{b} ± 9.09	222.6^{b} ± 8.09
Vitamin B_{12} (µg)	3.67 ± 0.21	2.89 ± 0.15	2.85 ± 0.21	3.79^{a} ± 0.22	2.91^{b} ± 0.17	2.69^{b} ± 0.16
Vitamin C (mg)	67.3^{a} ± 5.00	79.1^{b} ± 5.20	78.2^{ab} ± 5.68	81.0 ± 4.95	72.4 ± 5.61	70.2 ± 5.36
Vitamin D^{3} (µg)	4.31 (3.77, 4.92)	3.74 (3.33, 4.21)	3.42 (3.00, 3.91)	4.48 (3.99, 5.03)	3.67 (3.21, 4.19)	3.35 (2.94, 3.83)
Calcium (mg)	948.6 ± 41.2	888.9 ± 40.4	865.9 ± 51.8	1043^{a} ± 48.1	861.5^{ab} ± 42.9	791.1^{b} ± 36.4

Iron (mg)	9.10[a] ± 0.39	8.27[a] ± 0.33	9.91[b] ± 0.42	9.63 ± 0.38	8.26 ± 0.38	9.21 ± 0.38
Magnesium[3] (mg)	200.3[a] (190.6, 210.6)	198.3[b] (188.7, 208.5)	244.7[c] (229.0, 261.5)	249.6[a] (237.5, 262.4)	208.5[b] (198.4, 219.2)	183.1[c] (174.2, 192.5)
Phosphorus (mg)	1145 ± 40.6	1053 ± 35.4	1105 ± 46.6	1262[a] ± 43.1	1066[b] ± 41.4	962.6[c] ± 30.7
Potassium (mg)	1993[a] ± 63.4	1920[a] ± 60.3	2213[b] ± 82.2	2314[a] ± 69.9	1979[b] ± 67.5	1798[c] ± 59.1
Sodium (mg)	2185 ± 70.9	1978 ± 78.9	1953 ± 78.6	2228[a] ± 79.6	1927[ab] ± 85.6	1949[b] ± 60.2
Zinc[3] (mg)	7.46 (6.98, 7.98)	6.82 (6.38, 7.29)	7.24 (6.78, 7.74)	8.17[a] (7.77, 8.58)	6.96[b] (6.51, 7.44)	6.42[c] (6.11, 6.75)

[1]Data are presented as means ± SE unless otherwise indicated.
[2]Values with different letter superscripts within hPDI and lhPDI are significantly different from a comparison of tertiles, using the GENMOD procedure (to implement the generalized estimating equation approach to control for correlated outcomes among siblings), adjusted for energy intake, and followed by a Tukey's test for multiple comparisons.
[3]Values are geometric means (95% confidence intervals).
Abbreviations used: hPDI, healthful plant-based dietary index; lhPDI, less healthful plant-based dietary index; MUFA, monounsaturated fatty acids; PUFA, polyunsaturated fatty acids.
